# Improving Digital Mental Health Services With and for National Minority, Indigenous, and Refugee Youth in Norway: The InvolveMENT Multiphase Mixed Methods Research Project Protocol

**DOI:** 10.2196/64531

**Published:** 2025-12-24

**Authors:** Petter Viksveen, Eline Ree, Stig Bjønness, Ketil Lenert Hansen, Laia Meldahl, Lou Krijger-Plagnol, Clare Relton, Francesca Cornaglia, Siv Hilde Berg, Solveig Hodne, Jo Røislien, Karina Aase, Anita Salamonsen

**Affiliations:** 1 Department of Quality and Health Technology, Faculty of Health Sciences, University of Stavanger, Stavanger, Norway; 2 SHARE Centre for Resilience in Healthcare, Faculty of Health Sciences, Postboks 8600 Forus, Stavanger, 4036, Norway, 47 51832737; 3 Department of Public Health, Faculty of Health Sciences, University of Stavanger, Stavanger, Norway; 4 Regional Centre for Child, Youth Mental Health and Child Welfare North (RKBU Nord), Faculty of Health Sciences, UiT The Arctic University of Norway, Tromsø, Norway; 5 Wolfson Institute of Population Health, Queen Mary’s Medical Faculty, Barts and The London School of Medicine and Dentistry, Queen Mary University of London, London, United Kingdom; 6 School of Economics and Finance, The Faculty of Humanities and Social Sciences, Queen Mary University of London, London, United Kingdom

**Keywords:** digital services, Indigenous, mental health, national minority, Norway, refugees, youth

## Abstract

**Background:**

Worldwide, minority youth receive culturally sensitive mental health services less often than the majority peer population. In Norway, limited research exists on the mental health and service use among youth from national minority (Forrest Finns, Kven or Norwegian Finns, Jews, Roma, and Romani), Indigenous (Sámi), and refugee backgrounds. Although the Norwegian government provides a public communication channel for youth, including mental health information and support, digital services have not been adapted to meet the needs of these groups. There is currently no research to determine the use, acceptability, effectiveness, cost-effectiveness, and safety of these services for these youth.

**Objective:**

The main aim of the *InvolveMENT* project is to improve the mental health of national minority, Indigenous, and refugee youth. The project’s objectives are, for these groups of youth, to (1) determine the mental health and digital support needs and possible barriers to and facilitators of service use; (2) assess the use of and satisfaction with digital services to meet their mental health needs; (3) explore their perspectives on digital mental health services; (4) develop recommendations that can be used to adapt digital services to meet their needs and rights; and (5) assess the use, acceptability, satisfaction, effectiveness, cost-effectiveness, and safety of adapted services.

**Methods:**

The 4-year *InvolveMENT* project consists of four phases: (1) establishing a longitudinal cohort consisting of national minority, Indigenous, and refugee youth, using surveys to assess their mental health, well-being, digital support needs, use and satisfaction with digital services, and possible barriers to and facilitators of service use; (2) conducting qualitative interviews with minority youth to explore their perspectives and synthesizing data from phases 1 and 2 for a mixed methods analysis; (3) involving youth and health care and other professionals to develop proposals to adapt and improve the existing digital services; and (4) a randomized controlled trial and a qualitative study to evaluate the adapted services.

**Results:**

Cohort and qualitative study designs have been completed. Ethics applications have been approved, and recruitment to the cohort and qualitative studies has started.

**Conclusions:**

The *InvolveMENT* project has the potential to enhance the accessibility and quality of health care services and early interventions, reduce inequality in service provision for minority groups, and strengthen collaboration between youth, public, and research organizations. Through this, it has the potential to improve the mental health of youth from these groups. The findings might be transferable to other minority groups, both nationally and internationally.

## Introduction

### Background

Approximately 10% of youth worldwide are diagnosed with mental disorders [1]. There are mental health disparities among minority youth internationally, and these are affected by social determinants of health that are influenced by factors such as socioeconomic status, access to education and health care, and experiences of discrimination [2]. Youth from Indigenous and national minority groups have poorer mental health than the general population. For example, a systematic review found that Indigenous populations had suicide rates 20 times higher than non-Indigenous populations, with the highest rates in youth aged 15 to 24 years [3].

The Indigenous population in Norway is the Sámi. There is limited knowledge about the mental health of Sámi youth. During the period from 1970 to 1998, suicide rates among Sámi youth were twice those of the majority youth population in Norway [4]. Internationally, asylum-seeking and refugee youth have high rates of mental disorders, such as posttraumatic stress disorder, anxiety, and depression [5-8]. In Norway, up to 42% of asylum-seeking and refugee youth have been found to fulfill the criteria for psychiatric disorders [9]. A recent review reporting on the mental health of the Roma population in Europe found a higher proportion of mental disorders among children compared to the majority population and limited access to mental health care [10]. There is, however, no knowledge about the mental health among youth who belong to the following five national minority groups: Forrest Finns, Jews, Kven or Norwegian Finns, Roma, and Romani [11].

### Mental Health Care Service Use Among National Minorities, Indigenous, and Refugee Youth

The mental health care needs of national minority, Indigenous, and refugee youth in Norway have only been assessed to a limited extent [9,11,12]. Service use may be low, and dropout rates may be high. There are cultural and language barriers, difficulties in developing trust, marginalization of minority groups, a lack of immigrant staff and interpreters, and ineffective care coordination [13,14]. Limited effort has been made to adapt services to meet their needs, despite the right to health pursuant to the International Covenant on Economic, Social and Cultural Rights (Article 12), which was made into Norwegian law in 1999. Although the Sámi Norwegian National Advisory Unit—Mental Health and Substance Use offers services to support the Sámi population, youth more often seek help from friends, partners, or family members [12,15], and scientific knowledge about their digital service needs is lacking. Apart from a single hospital-based mental health clinic, the Transcultural Centre at Stavanger University Hospital [16,17], no clinics in Norway specialize in or have adapted services to meet the mental health needs of asylum-seeking and refugee youth. Studies that focus on the different cultural backgrounds of youth (ie, culturally sensitive studies) are needed to determine how to develop services that are culturally relevant and appropriate to support minority youth [18-20].

### Digital Mental Health Care Services for National Minority, Indigenous, and Refugee Youth

Young people often use the internet to access information about mental health [21]. According to the technology acceptance model, youth are more likely to use digital services if they perceive them as both useful and easy to use. Moreover, trust in technology plays a central role in youths’ engagement with digital services [22]. Digital mental health interventions may facilitate service access for national minority, Indigenous, and refugee youth by addressing geographic, economic, and social disparities [23,24]. However, there is an evidence gap, and culturally sensitive, effective, and safe modes of digital health care delivery must be further developed and tested [24,25].

*DigiUng* is a Norwegian public cross-sectoral collaboration, run by the *Directorate for Children, Youth and Family Affairs* and the *Directorate of Health*. It has developed the public communication channel *ung.no* to provide information, health care, and support services for youth. However, these services have not been adapted to meet the mental health needs of national minority, Indigenous, and refugee youth, and there is no research evidence regarding their acceptability, effectiveness, cost-effectiveness, and safety for these groups.

### Aim and Objectives

The overall aim of the *InvolveMENT* project is to improve the mental health of national minority, Indigenous, and refugee youth. This will be done by adapting digital mental health services to meet their personalized information and support needs.

The objectives of the *InvolveMENT* project are to (1) determine mental health and digital support needs and the barriers to and facilitators of service use for national minority, Indigenous, and refugee youth; (2) assess the use of and satisfaction with digital services to meet the mental health needs of national minority, Indigenous, and refugee youth; (3) explore the perspectives of national minority, Indigenous, and refugee youth on digital mental health services; (4) develop recommendations that can be used to adapt digital services to meet the needs and rights of national minority, Indigenous, and refugee youth; and (5) assess the use, acceptability, satisfaction, effectiveness, cost-effectiveness, and safety of the adapted digital services to meet the support needs of national minority, Indigenous, and refugee youth.

## Methods

### Study Design

The *InvolveMENT* project uses a participatory, multidisciplinary, multiphase, mixed methods research design [26] to adapt digital mental health services to meet the mental health needs of national minority, Indigenous, and refugee youth in Norway. The four stages of the research include a cohort study (work packages [WP1]) to assess the needs, barriers, and facilitators of service use; qualitative studies (WP2) to explore the perspectives of youth from these groups on digital mental health services; co-design activities (WP3) to develop recommendations for adapting digital services; and an evaluation study (WP4) to assess the adapted digital mental health services through a randomized controlled trial (RCT) and a qualitative study.

The project will use the Trials within Cohorts (TwiCs) design to test the acceptability, effectiveness, cost-effectiveness, and safety of the adapted digital services in WP4. This design offers several advantages over conventional RCTs and quasi-experimental approaches, particularly in studies involving hard-to-reach populations. TwiCs design enables fast and efficient recruitment to the RCT, as participants are already enrolled in the broader cohort from which trial participants are selected. It helps to reduce attrition rates because individuals in the control group are not informed about interventions they are not offered, thereby minimizing disappointment bias. Moreover, by avoiding disclosure of unavailable treatments, the design enhances the generalizability of results, as participants’ expectations and behaviors are less likely to be influenced by knowledge of the intervention [27]. One-year rolling recruitment is used to compensate for natural attrition.

A participatory, multidisciplinary, multiphase, mixed methods design is particularly suited to complex health interventions, as it allows for the integration of diverse perspectives and methodological approaches. The participatory component ensures that youth voices are actively included throughout the research process, enhancing cultural relevance, acceptability, and ethical rigor. The multidisciplinary and multiphase structure enables the project to move iteratively from needs assessment to service adaptation and evaluation, ensuring that findings from each stage inform subsequent phases. Finally, the mixed methods approach provides both the breadth of quantitative data and the depth of qualitative insights, offering a more comprehensive understanding than any single method alone.

A participatory design approach is used to adapt digital services [23], involving national minority, Indigenous, and refugee youth and professionals working in health services, social services, and related sectors (eg, education). Close collaboration, cultural sensitivity, and empowerment of participants are used to develop strong partnerships and to ensure co-ownership. The project encourages and facilitates multisectoral collaboration between the services and support and interest organizations for national minority, Indigenous, and refugee youth; health care and service providers; and university-based research centers. The collaborative component with active involvement of youth strengthens the relevance of the research to meet the needs of these groups of youth. Youth representatives, representatives of health and service providers, health care workers, and co-researchers from minority groups are involved in all phases of the project, including planning, interpretation, and dissemination of results [28].

### Study Setting or Location

This national project involves youth and professionals in all 4 health regions in Norway. Online questionnaire surveys are carried out in the cohort study (WP1) and the RCT (WP4), facilitating participation of youth from different parts of the country. The locations for the qualitative studies (WP2) and co-design workshops (WP3) vary for the different minority groups but are spread throughout the country.

### Study Population

Throughout all phases of the project, participants will be recruited among youth who identify as belonging to at least one of the following groups: national minorities (Forest Finns, Jews, Kven or Norwegian Finns, Roma, and Romani), Indigenous Sámi youth, and refugees. In addition, in the first 2 of 3 cohort surveys (WP1), youth not belonging to the mentioned minority groups will be included. The inclusion of the broader group of youth will enable comparisons between minority and nonminority youth. During the co-design phase (WP3), professionals working in health and social services who engage directly with these youth groups will also be recruited.

In WP1, up to 1500 youth with minority backgrounds will be recruited for each of the 3 cohort surveys. Additionally, the first 2 cohort surveys will include up to 1500 youth who do not identify as belonging to the described minority groups. WP2 will include 50 to 100 youth from the different minority groups, ensuring representation across all groups. WP3 will involve a minimum of 50 youth and professionals. WP4 will recruit approximately 300 youth from the WP1 cohort for an RCT and 25 to 45 youth for a qualitative study based on their exposure to the adapted digital services, regardless of RCT participation.

### Inclusion and Exclusion Criteria

In WP1, WP2, and WP3, the inclusion criteria are youth aged 16 to 25 years who identify as belonging to at least one of the following groups: national minorities (Forest Finns, Jews, Kven or Norwegian Finns, Roma, and Romani), Indigenous Sámi youth, and refugees. The term “refugees” is used inclusively to refer to all youth who consider themselves to be refugees, regardless of their official status as defined by the national government. Moreover, the first 2 cohort surveys in WP1 also include youth aged 16 to 25 years who do not define themselves as belonging to any of these minority groups. WP3 also includes professionals working in health services, social services, and related sectors (eg, education) who engage directly with youth from minority backgrounds, as well as youth representatives aged 16 to 35 years who belong to any of the mentioned minority groups. Additional inclusion criteria for youth participating in the RCT in WP4 include a Patient Health Questionnaire (PHQ-9) score indicating mild to moderately severe depressive symptoms (scores from 5 to 19). Youth included in the qualitative study in WP4 are those who have been exposed to the adapted digital services. Youth aged <16 or >25 years are excluded from participation. Participants across all groups must provide written informed consent to take part in the research.

### Study Outcomes

Given the nature of the *InvolveMENT* project, specific primary and secondary outcomes are defined for the RCT (WP4), which is the only component designed to evaluate the effectiveness of adapted digital mental health services quantitatively.WP2 and WP3 are exploratory and developmental in nature and therefore do not include predefined outcome measures. The primary outcome is the PHQ-9 score at 12 months of the trial. The secondary outcomes include scores for Generalized Anxiety Disorder [29,30], the Flourishing Scale [31], and the Children’s Somatic Symptoms Inventory 8 [32]. These outcomes are based on self-report and may be influenced by factors such as cultural, language, and social differences in diverse minority populations. While no single outcome measure has been validated, and culturally adapted tools are lacking across the included minority ethnic groups, these commonly used instruments have been selected as a choice to support knowledge generation in the absence of more inclusive and adapted alternatives. Service acceptability will be assessed by calculating the proportion of participants who use the adapted services.

### Study Procedures

#### Participant Recruitment

Participant recruitment in WP1, WP2, and WP3 is conducted through a broad range of channels tailored to reach minority ethnic youth from diverse backgrounds. These include secondary schools nationwide—particularly those with introduction classes for refugee youth and those with higher proportions of Indigenous and national minority youth—as well as higher education institutions (including one for students with Indigenous backgrounds), health care services, and language learning centers. Recruitment also involves collaborating with the national minority youth organizations, which are partners in the project, using their networks and social media platforms. In addition, outreach is conducted via social media channels commonly used by these groups of youth populations, such as TikTok (ByteDance), Instagram (Meta Platforms), and Snapchat (Snap Inc.). Recruitment takes place nationwide to ensure a diverse representation of perspectives. Participants who wish to contribute further may contact the research team to express interest in taking part in qualitative interviews and co-design workshops. A rolling recruitment method will be used over a period of 1 year for WP1 and WP4.

In the evaluation phase (WP4), participants for the RCT are selected from among those who scored between 5 and 19 on the PHQ-9 at baseline, indicating mild to moderately severe symptoms of depression. Participants for qualitative interviews in this phase are recruited based on their experience with the adapted digital mental health services.

#### Randomization

In WP4, participants who meet the inclusion criteria will be randomly allocated in a 1:1 ratio using Microsoft Excel, either to receive the intervention (the adapted digital services) or to continue with usual care as part of the control group. Those assigned to the control group will not be informed that they are part of a trial or that other participants are receiving an intervention. This approach is consistent with the TwiCs design [27,33], which stages and tailors the consent process to reflect real-world clinical practice. The design ensures that individuals receiving usual care are not informed about treatments they will not be offered, thereby minimizing potential for disappointment bias.

#### Data Collection, Interventions, and Follow-Up Procedures

As part of the cohort study (WP1), the project aims to recruit up to 1500 youth aged 16 to 25 years from national minority, Indigenous, and refugee backgrounds. On the basis of currently available data, it is reasonable to estimate that the population includes between 50,000 and 65,000 youth with minority ethnic backgrounds [34-40]. Data will be collected at 3 time points (ie, at baseline, 6 months, and 18 months) using online surveys developed in collaboration with youth minority representatives and partner organizations. The surveys are available in 14 languages: Arabic, Dari, English, Farsi, French, Kven, Lule Sami, Northern Sami, Southern Sami, Norwegian, Russian, Swahili, Tigrinya, and Ukrainian. Draft versions were piloted in English, Norwegian, Arabic, and Dari with a separate sample of 1500 participants, including youth from the majority Norwegian population and those with immigrant backgrounds [41,42]. The baseline survey will serve both as a primary data collection point and as a pilot phase to evaluate and refine recruitment strategies.

The qualitative studies (WP2) aim to explore the perspectives of minority youth on digital mental health services through individual and focus group interviews depending on participants’ preferences. These interviews will examine youths’ understanding of mental health; their needs for digital services, including those related to culture, language, and gender; and their pathways to support. They will also explore experiences with service availability, approachability, acceptability, and appropriateness, including trust in services and professionals. Participant numbers will be guided by the concept of information power [43]. Interviews will be conducted, whenever possible, in participants’ preferred languages, with professional interpreters used when necessary. Interview guides have been developed in collaboration with youth representatives and project partners. All interviews will be audio recorded and transcribed verbatim.

The co-design study (WP3), which will develop recommendations for adapting digital services to better meet the needs and rights of youth from national minority, Indigenous, and refugee backgrounds, will include youth recruited through the WP1 cohort and WP2 interviews, youth representatives, co-researchers, and professionals from health and social services (eg, school nurses, psychologists, general practitioners, child welfare workers, and employment office staff). A series of 6 to 8 workshops involving youth (approximately 4‐7), professionals (approximately 4‐7), or both will be conducted. However, the number and composition of workshops may vary, as they can be conducted with a single minority group or a mix of youth from different backgrounds, depending on participant preferences and the type of input needed to inform the recommendations. Data will be collected through observations and field notes and will be enriched by findings from WP1 and WP2, youth input from a dedicated seminar, and contributions from project meetings and events. Youth will be actively involved throughout the co-design process, from planning and recruitment to facilitation, ensuring their right to be heard at the systems level, in accordance with Article 12 of the UN Convention on the Rights of the Child [44]. The resulting recommendations will be presented to service providers to support informed decisions on implementation.

The evaluation study (WP4) will assess the implementation of recommendations developed in WP3 through two complementary procedures: an RCT and a qualitative study. The RCT will use the same survey instruments as WP1, with baseline data collected at the 6-month survey (before service adaptations) and follow-up data at the 18-month survey. The qualitative study will explore youth experiences with the adapted digital services, focusing on perceived support, safety, and satisfaction. Data will be collected through individual and focus group interviews depending on participants’ preferences. They will be conducted in participants’ preferred languages with professional interpreters as needed. Participants may include both RCT participants and others exposed to the adapted services. The number of participants will be guided by the concept of information power [43], with an estimated 25 to 45 participants. Interviews will be analyzed using qualitative content analysis [45], and findings will be integrated with RCT data through mixed methods analysis, applying triangulation to assess convergence, complementarity, and discrepancies [46].

#### Measurement Tools

Measurement tools in the cohort study (WP1) and the RCT (WP4) include self-report questionnaires, PHQ-9, the Generalized Anxiety Disorder scale [30], the Flourishing Scale [31], the Children’s Somatic Symptoms Inventory 8 [32], and single variables to determine participants’ familiarity with, use of, and satisfaction with digital services, as well as their perspectives on service providers’ cultural and linguistic competence and youths’ preferences regarding continuity and confidentiality in care.

#### Safety Considerations

The project includes several measures to ensure participant safety and well-being. Participants who may need support during the study are encouraged to contact established health care services, including school health professionals, general practitioners, and mental health helplines, with emergency services available for urgent situations. The project also includes culturally sensitive communication and interview procedures to reduce the risk of misunderstanding or harm.

#### Data Safety

All personal data collected are handled in accordance with strict privacy regulations. Data are used solely for the purposes outlined in the study, and participants’ confidentiality is safeguarded throughout. Participants also retain the right to access, correct, or delete their personal information and to file complaints with the Data Protection Authority if they have concerns about data processing. All sensitive data are securely stored on a dedicated server. These measures ensure that the research is responsive to participants’ needs and conducted with appropriate protections.

### Statistical Considerations and Data Analysis

#### Sample Size Calculation

Approximately 25% of youth are expected to experience mild to moderately severe symptoms of depression (PHQ-9 scores of 5‐19) [9,12,47]. To ensure a sufficient number of eligible participants for the RCT (WP4), a population cohort of 1500 youth will be recruited. This cohort size is designed to provide an adequate number of individuals who meet the inclusion criteria, which are scores of mild to moderately severe range on the PHQ-9, and to account for potential exclusions due to eligibility, nonconsent, or attrition. On the basis of an anticipated 20% dropout rate, 80% statistical power, a significance level of 0.05, and a moderate effect size of 0.35 (Cohen *d*), with equal allocation between intervention and control groups, the required sample size for the RCT is estimated to be 294. This has been rounded up to 300 to maintain sufficient power and accommodate potential variability.

#### Participant Numbers in the Qualitative Phases

In the qualitative phases of the project (WP2 and the qualitative component of WP4), participant recruitment will be guided by the principle of *information power*, which posits that the number of participants should be determined by the richness, relevance, and sufficiency of the data in relation to the study objectives [43]. For planning purposes, the estimated number is 50 to 100 youth participants for WP2 and 25 to 45 participants for WP4. However, these estimates remain flexible and will be adjusted based on ongoing assessments of data adequacy in addressing the research questions. Recruitment will also be shaped by practical constraints, particularly the limited availability of participants from certain minority youth populations, which may impact the final participant numbers.

#### Data Analysis

Quantitative data analyses will be carried out for WP1 and WP4. In WP1, data analysis will include univariate analyses for descriptive statistics, whereas factors associated with service use will be assessed using correlation analyses, regression models, and multiple regression models to explore multiple predictors and adjust for confounders, for example, generalized linear models and linear and generalized additive models for nonlinear associations between predictor and outcome. Temporal changes will be assessed using mixed models to assess factors affecting service use.

To assess the use and acceptability of the intervention, that is, the adapted digital mental health services for youth, we will use data from the RCT to determine the proportion of participants who report using it. An intention-to-treat analysis will be used to test the effectiveness of the intervention offer, whereas an instrumental variables analysis will be applied to determine the effectiveness of the received intervention [48]. The primary outcome will be the PHQ-9 at 12 months of the trial. Subgroup analyses will be carried out for different minority groups. Results will provide data on variability in depression outcomes in the different groups. Safety will be assessed by determining the proportion of participants with deteriorated mental health status through higher PHQ-9 scores. Service satisfaction will be assessed using single-item questions, each targeting participants’ satisfaction with 1 of 8 different types of digital services. A cost-effectiveness analysis of the intervention for youth will be carried out by undertaking a cost-consequences analysis [49]. It will compare individuals taking advantage of the adapted services with those who do not. The analysis will take a societal perspective, relating costs associated with introducing the new interventions and attributable to youths’ mental health, school absence, and work productivity (if they are in work), as well as their quality of life.

Qualitative data analyses will be carried out in WP2 and the qualitative part of WP4. The data will be analyzed using qualitative content analysis [45] or similar types of analyses. Moreover, 2 mixed methods analyses will be conducted: the first will integrate qualitative data from WP2 with quantitative data from the cohort surveys in WP1, using triangulation to assess convergence, complementarity, and discrepancies [46]; the second will be carried out within WP4, combining data from the RCT and qualitative interviews to evaluate the implementation and impact of adapted digital services. The specific analytic approaches for both mixed methods analyses will be determined based on the nature and quality of the data collected, allowing for flexibility to ensure methodological rigor and relevance.

Data collected in WP3 will be analyzed using a thematic approach, drawing on principles of participatory and design-based research. Field notes and observations from the co-design workshops will be reviewed to identify recurring themes, patterns, and insights related to youth and professional perspectives on digital service adaptation. These qualitative data will be triangulated with findings from WP1 and WP2, as well as input gathered during youth seminars and project meetings, to ensure a comprehensive and contextually grounded analysis. The analysis will be iterative and collaborative, involving youth coresearchers throughout the process to ensure that interpretations reflect their lived experiences and priorities. This participatory approach supports the development of actionable recommendations that are both evidence-informed and aligned with the rights of youth, particularly those from minority backgrounds. The final recommendations will be synthesized and presented to service providers for consideration and potential implementation.

### Ethical Considerations

According to the World Medical Association’s Declaration of Helsinki, vulnerable groups may participate in research when it is responsive to their health needs and priorities and when appropriate protections are in place. This project is supported by strong legal and ethical arguments, particularly because the voices of youth from minority backgrounds are seldom heard and they remain underrepresented in health research, despite existing public policies and legislation advocating for inclusion.

Participants are aged ≥16 years, granting them the legal right to independently decide on their participation. To ensure informed consent, participants receive both written and verbal information. Safeguarding procedures have been developed in collaboration with youth minority representatives and professionals working with these groups. These procedures include mechanisms for recommending additional health care support when needed, thereby protecting the well-being and integrity of participants.

While the researchers maintain overall responsibility for the project, it is designed as a collaborative effort involving multiple stakeholder groups. These include youth representatives from minority backgrounds, health care professionals, and service providers. Their involvement spans all phases of the project, from planning and design to data analysis and dissemination, to ensure cultural sensitivity and relevance. This includes co-development of research materials, such as information sheets, consent forms, interview guides, survey instruments, and promotional content. Such collaboration helps minimize the risk of misrepresentation or harm to minority youth participants. Stakeholders also contribute perspectives during data analysis and are invited to co-author scientific and popular publications, as well as participate in media and social media outreach. Written and signed collaboration agreements have been established to clarify the rights and responsibilities of all parties involved.

The project uses the TwiCs design, which asks potential participants to consent to the use of their identifiable data, to be recontacted, and to allow their data to be used in evaluating interventions [27]. Only those offered an experimental intervention are informed about the developed intervention, aligning the consent process more closely with real-world clinical practice. This approach is considered more ethical than traditional models that require full disclosure of all potential interventions upfront [33,50]. TwiCs studies have received ethical approval in 10 countries [33], including youth mental health research [51]. Youth participants were offered gift cards for their involvement in each activity, in appreciation of their time and contributions: NOK 100 (US $10) for surveys (WP1/WP4), NOK 250 (US $25) for qualitative interviews (WP2), and NOK 600 (US $59) for workshops (WP3).

The University of Stavanger, through SHARE—Centre for Resilience in Healthcare, holds overall responsibility for data management, with the project lead serving as data steward. The project adheres to General Data Protection Regulation (GDPR) [52] and Norwegian ethical standards [53], ensuring informed consent and secure handling of personal and sensitive data. Consent procedures include options for recontact and data sharing, with safeguards for anonymity and participant rights. All data handling, including classification, storage, and processing, follows strict protocols to ensure privacy, integrity, and ethical compliance throughout the project lifecycle. Sensitive data are stored on a Sensitive Data Services server.

Ethical approval for this project has been granted by the Regional Committees for Medical and Health Research Ethics (ID 780840). Additionally, collective consent for Sámi youth participants has been approved by the Expert Ethics Committee for Sámi Health Research (ID 1133459). Moreover, Sikt, the Norwegian Agency for Shared Services in Education and Research, has considered the processing of personal data to be lawful based on consent (GDPR Article 6(1)(a)) and explicit consent (GDPR Article 9(2)(a), applicable to special categories of data) [52] (reference number 374832).

## Results

We have completed recruitment to the project of youth representatives from all national minority, Indigenous, and refugee youth groups, which are the end users of the research. The panel now consists of 32 youth representatives. Ethics approval has been granted. A recruitment plan has been developed, and recruitment is estimated to start in December 2024. Draft versions of the cohort survey have been piloted in English, Norwegian, Arabic, and Dari, with a separate sample of 1500 participants [41]. This pilot study was conducted before the main data collection and included youth from both the majority population in Norway and those with immigrant and refugee backgrounds. On the basis of feedback from the pilot, the survey was subsequently adapted in collaboration with youth minority representatives and other project partners. This cohort study has been translated into 14 languages and will include up to 1500 youth who identify as belonging to national minority, Indigenous, or refugee backgrounds. In addition, another 1500 youth who do not belong to these groups will be included to enable meaningful comparisons between minority and nonminority populations. Youth minority representatives have also contributed input to the planning of qualitative studies. Funding was obtained from the Research Council of Norway for the time period from July 1, 2023, to June 30, 2027. Data collection started in January 2025, after submission of the manuscript (November 18, 2024).

## Discussion

### Anticipated Findings

The *InvolveMENT* project will offer an understanding of the mental health status of youth with national minority, Indigenous, and refugee backgrounds, together with their familiarity with and use of digital mental health services. This will supplement the existing research evidence, which only to a limited extent reports on perceptions of the impact of digital services on mental health among groups of minority youth [54]. While existing platforms provide general access to support, they often fall short in addressing the culturally specific requirements of these populations. Together with language needs, this can undermine youths’ trust in service providers and ultimately serve as a barrier to the use of services [24].

Adapting digital mental health services also holds the potential to address challenges resulting from refugee youths’ limited knowledge about the national health system and to reduce the harmful effects of mental illness–related stigma [55]. Through both survey data and qualitative interviews, the project will explore how youth from diverse backgrounds understand mental health, their experiences with digital services, and their preferences for culturally relevant support. These insights will inform the co-design process involving minority youth and professionals working closely with them, resulting in concrete proposals for culturally adapted, equitable, and sustainable digital mental health services.

The final phase will bring new knowledge about the acceptability, effectiveness, cost-effectiveness, and safety of digital services aimed at meeting the needs of minority ethnic youth in Norway. Others have stressed the importance of integrating user experiences to assess acceptability, self-reported effectiveness, and satisfaction with services [24]. This phase will lay the groundwork for future trials by identifying variability in mental health and service needs across different groups.

An expected impact of the project is to increase the proportion of minority youth who seek and receive appropriate digital support at an early stage, thereby contributing to timely diagnosis, intervention, and prevention of prolonged mental health complaints. As found by others, digital mental health interventions have the potential to support youth with refugee backgrounds who may be reluctant to consult mental health professionals due to fear or shame [24].

Moreover, by establishing a cohort using the TwiCs design, the project also creates opportunities for testing additional interventions during and beyond the 4-year study period. The project will also expand international research networks and explore the development of future collaborative studies focused on minority youth in diverse global contexts.

### The Involvement of Youth Throughout the Research

The *InvolveMENT* project marks the first time national minority, Indigenous, and refugee youth—whose voices have rarely been heard—are actively involved in assessing and developing digital mental health services. Research using participatory approaches, which actively engage youth and integrate cultural elements into interventions, has been found to facilitate the development of programs that meet the needs of minority youth, such as those from Indigenous backgrounds [56]. It is our hope that the extensive involvement of youth in the project may also contribute to reducing concerns about privacy and confidentiality, which are common among these groups of youth [57]. Together with the involvement of professionals providing services for these groups of youth, the extensive and active involvement of youth in co-design processes ensures that their needs remain at the center of the research aimed at the development of adapted, equitable, and sustainable health care support services.

### Strengths and Limitations

The strengths of the *InvolveMENT* project include the novelty of the project, which aims to improve mental health outcomes for national minority, Indigenous, and refugee youth in Norway by addressing the lack of culturally sensitive digital services tailored to their needs. The broad range of methodologies used will contribute to a better understanding of youth from minority ethnic groups, in particular with regard to the development of culturally adapted services that are suited to meet their mental health needs. A key strength lies in the youth-centered approach, ensuring that services are equitable, relevant, and sustainable, and marking the first time these populations are directly engaged in shaping mental health support in Norway. The main risk of this project is the recruitment of national minority, Indigenous, and refugee youth for the cohort (at least 1500 youth) and for the qualitative study. There are no exact population figures for the included minority groups, but estimates suggest 50,000 to 65,000 (age group 16‐25), and recruitment will take place through multiple channels. A recruitment rate of 2% to 3% is a conservative estimate as participation rates in youth surveys were 73% to 87% in the Norwegian *Ungdata* survey [58] and 77% in our pilot [42]; and minority youth will be informed that the project aims to hear their views and that they will influence service development, which will encourage participation. The substantial involvement of youth representatives and coresearchers from minority groups in the project will be a great advantage for the recruitment process. Another limitation is the reliance on self-reported measures, which may introduce bias due to cultural differences in language and response styles. This concern may be particularly relevant in culturally diverse populations, where language, response styles, and social desirability norms can influence how questions are understood and answered [59]. However, the randomized design enhances internal validity, and the integration of qualitative interviews with survey data allows for triangulation that may help mitigate these limitations. By assessing the acceptability, effectiveness, cost-effectiveness, and safety of adapted services, the project not only enhances early intervention and reduces long-term mental health issues but also lays the foundation for future trials and international collaboration. Its findings will generate vital knowledge, expand research networks, and offer scalable models for culturally adapted digital mental health services worldwide.

### Dissemination Plan

The project’s dissemination strategy includes a variety of communication and publication activities. It encompasses a diverse range of dissemination activities tailored to various audiences. These include publishing scientific articles in journals and popular science outlets to engage the scientific community, professionals, youth, and interest organizations; and producing project reports and academic theses aimed at public decision-makers. Additional outreach efforts involve media interviews and articles for youth, parents, schools, and the general public; oral and poster presentations at conferences and seminars; contributions to organizational publications; and dynamic content on social media platforms such as TikTok, Snapchat, LinkedIn, Instagram, and Facebook, designed to reach youth, parents, interest organizations, and the broader public.

### Conclusions

The *InvolveMENT* collaborative research project will contribute to improving the quality, equity, and efficiency of digital mental health services for groups of minority youth in Norway. The project will assess their use of and experiences with digital mental health services. Services can be adapted so they are culturally sensitive and meet the needs of national minority, Indigenous, and refugee youth.

## Supplementary material

10.2196/64531 Checklist 1 NALHN Research Governance Office - Research Protocol Template

## Abbreviations

CCA: cost-consequences analysis
CEA: cost-effectiveness analysis
CSSI-8: Children's Somatic Symptoms Inventory
GDPR: General Data Protection Regulation
ITT: Intention-To-Treat
IV: instrumental variables
PHQ-9: 9-item Patient Health Questionnaire
RCT: randomized controlled trial
TwiCs: Trials within Cohorts
WP: work packages
