# Supplementary material for: Improving Digital Mental Health Services With and for National Minority, Indigenous, and Refugee Youth in Norway: The InvolveMENT Multiphase Mixed Methods Research Project Protocol
Source: JMIR Res Protoc. 2025 Dec 24;14:e64531. doi: 10.2196/64531 (PMC12736645; doi:10.2196/64531)
Supplement: Checklist 1 [file resprot-v14-e64531-s001.pdf]

NALHN Research Governance Office - Research Protocol Template
Available at URL
https://www.sahealth.sa.gov.au/wps/wcm/connect/public+content/sa+health+internet/resources/nalhn+rgo+-+research+protocol+template

Note: The page numbers shown in these guidelines, which reference information from the research protocol entitled *"Improving Digital Mental Health Services With and for National Minority, Indigenous, and Refugee Youth in Norway: The InvolveMENT Multi-Pphase Mixed Methods Research Project Protocol"* [JMIR Res Protoc 64531] submitted for journal publication, are highlighted in yellow for ease of identification.

A well written and complete protocol is essential for a high quality research project. A study protocol generally follows a conventional layout. There are several templates already available although most are developed for commercially-sponsored randomised controlled studies. This research protocol template aims to offer Mater medical, nursing, midwifery and allied health researchers a more generic guide suitable for a broader range of clinical and scientific research enquiries.

The preparation of a protocol is an important first step in the research process for the following reasons:

1. It states the research question you aim to answer;
2. It encourages adequate consideration and planning of project detail *before* you begin;
3. It allows co-investigators or peers a living and dynamic document for contribution and early review prior to its completion;
4. It acts as a record and reminder for you and your supervisor (co-investigator or co-worker) of the initial project aims and stated procedures. This record also enables you to monitor the progress of your project; and
5. It provides the basis for funding or human research ethics applications.

The template contains a broad outline of sections usually expected in a research protocol. It is a guide to the information conventionally required rather than aiming to be definitive. Therefore, not all of the sections will be relevant for every study protocol and may be modified or deleted as applicable.

STUDY TITLE Page 1

A well constructed study title is important as it is the first opportunity to attract the attention of the reader. The study title should be descriptive, although clearly and concisely indicating the subject of inquiry. Having a refined research question can assist in constructing a title. This will ensure that your study title reflects (if appropriate) the patient population, intervention (e.g. medicinal product or device), comparator (e.g. another intervention, placebo or usual care) and outcome. You might also consider incorporating the design type (e.g. a randomized controlled study, case-control study, or retrospective cohort study) as is recommended to improve the *reporting* of health research (e.g. Consolidated Standards of Reporting Studies, or CONSORT). However, your initial title will only be a working title that would usually be revised as your study becomes more refined. The final title should be consistent across all related documents (including regulatory documents if applicable). You might also like to include a 'lay' ('public' or 'simplified') title easily understood by non-medical or interdisciplinary persons and/or an acronym. These are sometimes asked for in funding applications, Human Research Ethics Committee (HREC) submissions, and clinical study registries.

e.g. "A randomized controlled study to evaluate the effect of tight glycaemic control in intensive care patients on survival".

STUDY INVESTIGATOR(S) Page 1

Principal Investigator (A):	Ms Iman U Researcher[1]<br>Ph. 3163 xxxx<br>Email: iresearcher@mmri.mater.org.au
Principal Investigator (B):	xxxxx[2]
Co-Investigator (A):	xxxxx[1]
Co-Investigator (B):	xxxxx[2]
Co-Investigator (C):	xxxxx[2]

1. Institution, street, suburb, city, state , country, etc.
2: Institution, street, suburb, city, state , country, etc.

1. INTRODUCTION Page 2

The introduction is a very brief overview of study (*~250 words*). The introduction should be concise but sufficient to orientate the reader to the main purpose of the study, how it will be conducted and its expected benefits. It is a structured sketch of the study that will provide an overview before examining the details. It is placed at the head of the protocol but is often written after the protocol itself is completed.

2. BACKGROUND Page 2

The most important aspect of a research proposal is the clarity of the research problem.
This is an opportunity to convince the reader (or reviewer) of why the study needs to be done (and deserves funding or ethical approval). Keep this brief and to the point (approximately two A4 pages). The following key points may be used as a guide:

- Conduct a comprehensive literature search (Cochrane, Medline, Embase and other databases relevant to your area of study). The UQ/Mater McAuley Library is a valuable resource for researchers on campus for assistance or advice on developing an optimal search strategy. The library also offers a literature search service for staff registered to use library services.
- Discuss the importance of the topic (public health and/or clinical importance and impact on individuals/community; incidence, prevalence, mortality and morbidity).
- Critically appraise the relevant literature and discuss the state of current knowledge on the topic (including deficiencies in knowledge or gaps that make the study worth doing).
- Indicate how the research question has emerged and fits logically with the above.
- Outline your approach to address the research question.
- Explain how your study will contribute to existing research and benefit other individuals or the wider community.

Discussion should be clear and logical that demonstrates you are fully conversant with the ideas presented and can grasp their methodological implications. Aim to be concise and present only key sources rather than an exhaustive list of cited references (limit to approximately 20-25 key papers). The literature review should logically lead to the statement of the aims of the proposed project.

3. AIM(S) OF STUDY Page 2

Your aim(s) should arise from your literature review and state what the study hopes to accomplish.

4. OBJECTIVES Page 3

Your focused research question needs to be further refined into one or more study *objectives*. The study objective(s) should be single and quantifiable statement(s) that will allow you to answer your research question.

e.g. The objective of this study is to determine if socioeconomic status is associated with excess childhood asthma in Istanbul.

5. HYPOTHESIS Not applicable

5b. Primary Hypothesis Not applicable

Hypotheses are more specific than objectives and amenable to statistical evaluation. Your primary hypothesis is your statement of the hypothesised effect on the primary outcome measure. A hypothesis is worded very simply and written as 'testable' statements. Your experimental results will prove or disprove your hypothesis. Hypotheses are generally stated in the null form (H_o) as they have their basis in inferential statistics. Rejecting the null hypothesis increases our confidence, with a given level of probability, that there is a relationship between the variables being studied. However, a classic scientific hypothesis includes both a null and alternative (H_a) hypothesis.

e.g. H_0: Asthma prevalence rates are not different among children from low and high socioeconomic groups in Istanbul.

H_A: Asthma prevalence rates are different among children from low and high socioeconomic groups in Istanbul.

5b. Secondary Hypotheses Not applicable

Although a study is usually based around a primary hypothesis, secondary hypotheses may also be pre-specified although based on outcomes of lesser importance or additional interest. As the primary hypothesis is usually the basis for statistical power calculations, secondary hypotheses with insufficient power will generally not lead to statistically robust conclusions.

6. STUDY DESIGN Page 3

State the design of the research (e.g. randomised controlled study, cross-sectional survey, prospective or retrospective cohort/case-control). Whatever the study design, you need to ensure that you provide the reader with a clear statement and description of your proposed design. You may also explain why the particular study design has been chosen in preference to other possible designs (i.e. justification for choice of study design).

7. STUDY SETTING/LOCATION Page 3

The location of where the study will be conducted (e.g. Neonatal Intensive Care Unit, Mater Mothers' Hospital). You need to mention whether the study is going to be a single-centre study or a multi-centered study (i.e. conducted in more than one location).

8. STUDY POPULATION Page 3

Defining the group in which the study will be carried out on provides the setting for which the research has relevance. This section also describes how one can be certain that the results from your sample population can be generalised to the target population of interest. This section should describe the target population, including:

- Population the subjects will be drawn from
- All aspects of subject selection
- The total number and number within any subgroups

9. ELIGIBILITY CRITERIA Page 4

Inclusion and exclusion criteria are standards that you have set determining whether a person may or may not be allowed to enter your study. They are used to identify appropriate participants and to ensure their safety.

9a. Inclusion criteria Page 4

Inclusion criteria are the 'characteristics' that clearly describe the study population that are required for a subject to be included in the study. The criteria may be based on factors such as age, gender, the type and stage of a disease, previous treatment history, and co-morbid medical conditions. They may state appropriate criteria for admitting special 'at-risk' populations such as women of reproductive age, children or patients with disease states or organ impairment.

9b. Exclusion criteria Page 4

Provide details of participants that will be considered ineligible to participate and justification for their exclusion. These criteria are not always clinical in nature, aiming principally to accommodate participants in a safe and ethical manner. Criteria may include circumstances that interfere with the participant's ability to give informed consent (diminished understanding or comprehension, or a language other than English spoken and an interpreter unavailable), contraindications to the study treatment(s)/procedure(s), taking certain concomitant medication(s), or conditions that interfere with a patient's ability to comply with all treatment(s)/procedure(s).

10. STUDY OUTCOMES Page 4

10a. Primary Outcome Page 4

The primary outcome should be the most important and clinically relevant outcome (e.g. clinical, psychological, economic, or other) of the study. This is the measure used to answer your study aim. However, it is also the outcome used to calculate study sample size and power and test the primary research hypothesis. Generally, no

more than 1-2 primary outcome measures are pre-specified. Primary outcome measures may be measured in various ways such as: binary (e.g. caesarean/no caesarean, blood loss ≥500mL/blood loss <500mL); continuous (e.g. weight - kg, blood loss - mL); ordinal (e.g. pain - mild, moderate, severe); time to event (e.g. survival), and counts (e.g. number of infections, number of events occurring).

10b. Secondary Outcome(s) Page 4

Secondary outcome(s) are measures of additional or less important research interest. They may include additional clinical, psychological, economic, or safety outcomes (e.g. treatment related side effects/adverse events). However, as these endpoints are not used to calculate study power and sample size it is often not possible to draw robust conclusions from the results.

11. STUDY PROCEDURES Page 4

This section should describe exactly what is going to happen during conduct of the study. It is preferable to use the active voice and state in the future tense (e.g. "We will randomly allocate subjects to…").

11a. Recruitment of participants Page 4

This section should describe which potential participants will be identified/selected for recruitment (e.g. via outpatient clinic, medical records search), how they will be approached/invited to participate and how consent will be obtained. You may need to justify the feasibility of recruiting the required number of subjects and estimate the proportion that you would expect will agree to participate. Finally, the period of time expected to recruit the required number of participants should be stated here.

11b. Randomisation Page 4

Include the method (including any software) used to generate the random allocation sequence. Describe type of randomisation performed, ratio of assignment to groups, block size permutation and stratification if applicable. Explain the methods used to conceal group allocation until assignment. Also, include information on who will generate the allocation sequence and who will assign participants into their groups.

This section should also discuss if participants, the investigator, and those assessing/analyzing the outcome(s) will be blind (or masked) to group assignment or if the study will be an open-label study (investigators and subjects know their assigned group).

11c. Study procedure Page 4-5

In this section you need to clearly and comprehensively describe exactly what will happen to participants once they are enrolled in your study. Depending on the study it might include how potential participants will be approached, when they will be randomised, the frequency and duration of visits or whether they are expected to self-complete a daily diary at home, the duration of the study or follow-up, and any measurements taken at each visit (e.g. questionnaires, physical measurements, biological samples).

You should include precise details of the treatment(s)/intervention(s) intended for each group/participant You should also provide details of any follow-up schedule (i.e. time between visits) and consider how you will monitor participants' adherence with the treatment schedule. You might also describe under which circumstances participants may be withdrawn and how this will occur. A schematic diagram or flow chart may be useful for this section.

For drugs and devices that are commercially available, the protocol must state their proprietary names, manufacturer, chemical composition, dose, duration and frequency of administration.

For drugs and devices that are still in the experimental stage (or commercially available and used for a different indication or mode of administration), an Investigators Brochure (IB) is a required accompanying document to the protocol. The IB is a compilation of clinical and non-clinical data, available pre-clinical experiments in animals and/or results of Phase I/II clinical studies available on the experimental products intended for use in the study in question. It provides study organisers and staff with an understanding of the rationale of the study in order to inform their compliance with the protocol requirements. In these cases the approval of the Therapeutic Goods Administration(TGA) will be required prior to commencing the study. Refer to the TGA website, for further information on submission requirements – www.tga.gov.au).

11d. Measurement tools used Page 5

It is essential to state how the data will be collected to assess the primary and secondary outcome(s) of the study (e.g. patient questionnaire, medical charts, routinely collected hospital/research database, biological specimens). Describe at what point(s) of the study data collection will occur. You should make statements that justify the validity of the study measure/instrument. If not, you will have to verify how you will ensure the validity and quality of data being collected. Also, mention here if you are going to have one or more assessors to collect data, their level of training/experience (or how they will be trained), and if you are planning to assess inter-rater reliability (if applicable).

11e. Safety considerations/Patient safety Page 5

The safety of research participants is foremost. You will need to provide adequate information on how the safety of research participants will be ensured. This can include procedures for recording and reporting adverse events (and serious adverse events) and their follow-up (mandatory requirement for studies involving intervention or treatments). Remember that even administering a research questionnaire may have adverse psychological effects on susceptible individuals.

11f. Data monitoring (this section can flow on from the one above providing more details on data monitoring and other quality control measures) Page 5

This section includes information on the personnel and processes of the Data and Safety Monitoring Committee, the use of study monitors to audit study conduct, any stopping and discontinuation rules pre-specified, and handling of adverse events/serious adverse events.

12. STATISTICAL CONSIDERATIONS AND DATA ANALYSIS Page 5-6

12a. Sample size and statistical power Page 5-6

A sample size or power calculation should be performed. This calculation is used to estimate the number of subjects required to answer your primary study hypothesis with an accepted power. Conversely, it also allows you to estimate what power can be achieved with a limited number of participants. This number is calculated by specifying the magnitude of the effects that are expected (i.e. informed and clinically significant), variability of the measurements and the acceptable degree of type I and II errors. You need to specify the assumptions made for the calculation. It is recommended that you consult with a statistician for this section. Also keep in mind the estimated recruitment rate and whether you need to adjust for anticipated non-responders and losses to follow up.

12b. Statistical methods Page 6

The statistical methods used for the study objectives/hypotheses (e.g. t-test, chi-squared, multivariate modeling) must be sufficiently detailed. If conducting a randomized controlled study, you should state whether methods will include an “intention to treat” (ITT) analysis, per protocol analysis, or both. An ITT analysis is preferred as it compares all subjects in the groups to which they were originally randomly assigned (despite withdrawal, treatment failure or cross-over). Consultation with a statistician is strongly recommended.

13. ETHICAL CONSIDERATIONS Page 6-7

You must state that the study will be conducted in full conformance with principles of the “Declaration of Helsinki”, Good Clinical Practice (GCP) and within the laws and regulations of the country in which the research is conducted. You will need to consider and articulate how the quality of the technical aspects have been assured, the potential risks and proposed benefits of the study procedures, the priority of the participants’ interests over those of science or of society and how those interests will be safeguarded, responsibility for liability of injury during the study, how the participants are informed of the study, and how they give voluntary consent to participate.

Information on how informed consent is to be obtained should be included. This ensures that if participants can read and understand the information they need to make an informed decision about their voluntary participation. This can include allowances for special population groups (e.g. children, Aboriginal and Torres Strait Islander) where applicable.

You will also need to adequately detail methods of data extraction (non-identifiable, de-identified or re-identifiable), and data management, storage and security storage (of paper hardcopies and/or electronic files).

For further information see the National Statement on Ethical Conduct in Human Research (NHMRC, 2007) and see the Mater Human Research Ethics Coordinator for advice and guidance on your particular study.

14. OUTCOMES AND SIGNIFICANCE Page 7-8

It may be of value to reiterate the potential benefits of answering the research question and conducting the project. This section restates the justification for the study in terms of the anticipated results. It may be important to specify the implications of the potential results and how the results of this study may inform future research or policy makers.

15. REFERENCES Page 9-12

World Medical Association Declaration of Helsinki (1964)
Note for guidance on good clinical practice (June 2018)
National Statement on Ethical Conduct in Human Research (2007) – Updated 2018
